# Rapid design and development of CRISPR-Cas13a targeting SARS-CoV-2 spike protein

**DOI:** 10.7150/thno.51479

**Published:** 2021-01-01

**Authors:** Lin Wang, Junhu Zhou, Qixue Wang, Yunfei Wang, Chunsheng Kang

**Affiliations:** 1 Department of Neurosurgery, Tianjin Medical University General Hospital, Tianjin 300052, China;; 2 Tianjin Neurological Institute, Key Laboratory of Post-neurotrauma Neuro-repair and Regeneration in Central Nervous System, Ministry of Education and Tianjin City, Tianjin 300052, China

**Keywords:** CRISPR-Cas13a, coronavirus, SARS-CoV-2, bioinformatics, RNA-editing

## Abstract

The novel severe acute respiratory syndrome coronavirus 2 (SARS-CoV-2) has caused a worldwide epidemic of the lethal respiratory coronavirus disease (COVID-19), necessitating urgent development of specific and effective therapeutic tools. Among several therapeutic targets of coronaviruses, the spike protein is of great significance due to its key role in host invasion. Here, we report a potential anti-SARS-CoV-2 strategy based on the CRISPR-Cas13a system.

**Methods**: A comprehensive set of bioinformatics methods, including sequence alignment, structural comparison, and molecular docking, was utilized to identify a SARS-CoV-2-spike(S)-specific segment. A tiling crRNA library targeting this specific RNA segment was designed, and optimal crRNA candidates were selected using *in-silico* methods. The efficiencies of the crRNA candidates were tested in human HepG2 and AT2 cells.

**Results**: The most effective crRNA sequence inducing a robust cleavage effect on S and a potent collateral cleavage effect were identified.

**Conclusions**: This study provides a rapid design pipeline for a CRISPR-Cas13a-based antiviral tool against SARS-CoV-2. Moreover, it offers a novel approach for anti-virus study even if the precise structures of viral proteins are indeterminate.

## Introduction

Severe acute respiratory syndrome coronavirus 2 (SARS-CoV-2) has caused a global pandemic of coronavirus disease 2019 (COVID-19). There are currently no specific therapeutic agents against SARS-CoV-2. Antiviral drugs, including lopinavir, ritonavir, remdesivir, and chloroquine, showed activity against SARS-CoV or SARS-CoV-2 *in vitro* 1, 2. However, recent clinical trials revealed that they provide limited benefit with respect to the clinical outcomes of COVID-19 patients 3, 4. Corticosteroid treatment is not recommended based on clinical evidence because of limited benefit and severe complications 5. Convalescent plasma or immunoglobulins exhibit therapeutic potential, but their safety and efficacy remain to be verified in large-scale clinical trials 6. Therefore, there is an urgent need for specific and effective therapies for this life-threatening disease.

LwaCas13a, an RNA-guided clustered regularly interspaced short palindromic repeats (CRISPR) effector that targets and cleaves single-stranded RNA (ssRNA), has gained increasing attention as a therapeutic and diagnostic tool in viral diseases and cancers. The CRISPR-Cas13a system is composed of two components: the RNA-guided RNase Cas13a and CRISPR RNA (crRNA) as the targeting single-guide RNA. Unlike other Cas nucleases, Cas13a exhibits a collateral cleavage effect, which means that after recognizing its target RNA, activated Cas13a not only cleaves target RNA, but also nearby non-target RNAs. Recent studies have highlighted the application of Cas13a in RNA knockdown, RNA detection, and RNA capture 7, 8. Zhang et al. developed a Cas13-based diagnostic tool, specific high-sensitivity enzymatic reporter unlocking (SHERLOCK), to detect viruses such as the Zika virus and dengue virus 9.

Our previous study demonstrated that the CRISPR-Cas13a system could exert collateral cleavage effects in human glioma cells 10. The catalytically dead variant of Cas13 (dCas13), generated due to mutations in conserved catalytic residues, can process crRNA and specifically bind target RNA, but loses its ability of RNA cleavage. Generally, dCas13 is utilized in RNA imaging and tracking and, when fused with the catalytic domain of human adenosine deaminase acting on RNA 2 (hADAR2d), is applied in programmable single-base RNA editing 11. Therefore, CRISPR-Cas13a may be a promising tool against RNA viruses.

SARS-CoV-2 is a positive-sense single-stranded RNA virus with an envelope. Its RNA genome (~30 kb) encodes viral proteases, RNA polymerase, and structural proteins, including spike (S), membrane (M), envelope (E), and nucleocapsid (N) proteins. The S protein is critical in mediating virus entry into host cells through interaction with surface receptors 12. Angiotensin-converting enzyme 2 (ACE2) has been demonstrated to be the host entry receptor for SARS-CoV and SARS-CoV-2 13, 14, and dipeptidyl peptidase 4 was identified as the receptor for MERS-CoV 15. Given that the S protein plays a key role in the induction of neutralizing antibody and protective immune responses, it is a significant epitope for pharmaceutic design 16, 17.

We employed a set of comprehensive bioinformatics methods to identify a unique segment in SARS-CoV-2 S, distinct from the corresponding segment in SARS-CoV S. Using this unique segment as a specific target for SARS-CoV-2 S, we designed a tiling crRNA library and selected crRNAs with GC contents above 0.5. Subsequently, molecular docking simulation was performed to analyze interactions among the crRNA candidates, target RNA, and Cas13a. Docking scores were calculated to evaluate crRNA-RNA-Cas13a complex stability, and crRNAs with lower docking scores were considered as potential optimal crRNAs. The editing efficiency and collateral cleavage effect of the crRNA candidates were evaluated in HepG2 human hepatocarcinoma cells and human alveolar epithelial type II (AT2) cells. Based on the comprehensive evaluation of crRNA candidates, crRNA-6 exhibited significant and stable efficacy in guiding cleavage. Our study provides a roadmap for the customized design of a CRISPR-Cas13a-based tool against SARS-CoV-2 and other coronaviruses.

## Results

### Customized design of a CRISPR-Cas13a system targeting and cleaving SARS-CoV-2 RNA

**Figure 1:**
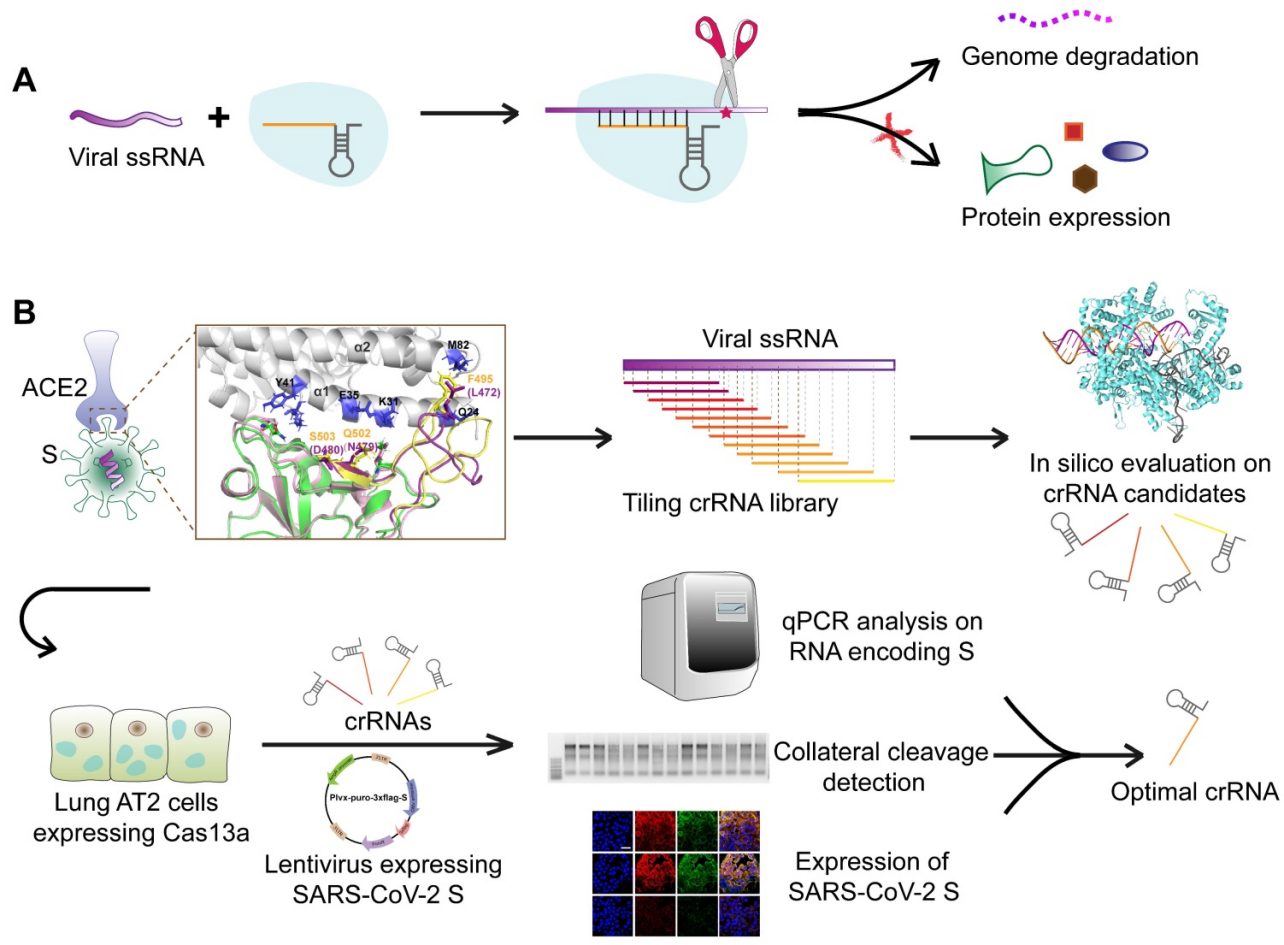
Schematic diagram of the design of the SARS-CoV-2-targeting CRISPR-Cas13a-based tool. A. The cleavage mechanism of CRISPR-Cas13a complex on viral single-strand RNA. The complementary segment in the crRNA sequence targets specific ssRNA and the hairpin-like non-complementary segment is involved in the binding of the crRNA to Cas13a. Following the activation of Cas13a, the target viral RNA is cleaved. The collateral cleavage effect results in the degradation of the viral genome and non-target mRNAs. B. Schematic representation of the identification of a specific SARS-CoV-2 target, and the design and screening of crRNA candidates. Bioinformatics analyses were used to identify a specific RNA segment in SARS-CoV-2 S, followed by designing a tiling crRNA library targeting this specific RNA segment. *In-silico* evaluation was performed to screen potential optimal crRNA candidates. The S RNA knockdown efficiency and collateral cleavage effect were examined in HepG2 and AT2 cells to identify an optimal crRNA.

We aimed to design an antiviral tool based on the RNA-editing function of the CRISPR-Cas13a system. Cas13a is activated by a specific crRNA that targets a specific single-stranded RNA sequence, leading to the cleavage of the target RNA and collateral cleavage. The specific crRNA targeting SARS-CoV-2 RNA activates Cas13a, resulting in the cleavage of the viral genome and blockage of viral protein synthesis (**Figure 1A**). We adopted a set of comprehensive methods to design a CRISPR-Cas13a-based tool specifically against SARS-CoV-2. The design was guided by bioinformatics and tested in HepG2 and AT2 cells (**Figure 1B**).

### Identification of a unique sequence segment in SARS-CoV-2 S receptor-binding domain (RBD)

**Figure 2:**
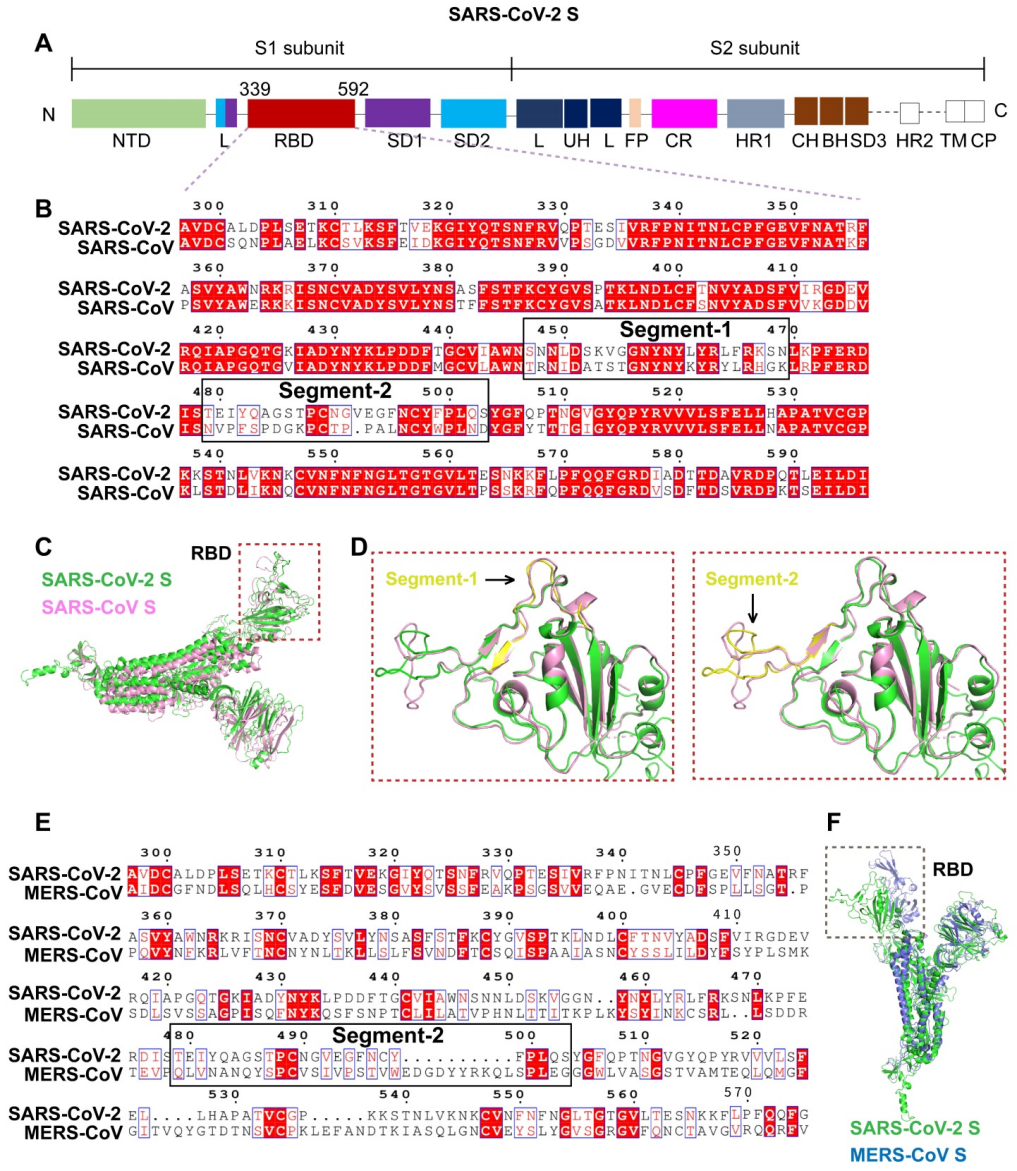
Identification of a unique segment in the SARS-CoV-2 S RBD. A. Schematic diagram of the protein domains of SARS-CoV-2 S. The RBD, comprising residues 339-592 of the S1 subunit, is a key region mediating virus-cell receptor interaction. B. Sequence alignment of SARS-CoV-2 RBD and SARS-CoV RBD revealed two segments with numerous residue differences. C. 3D-structural superimposition revealed the high structural homology between SARS-CoV-2 S and SARS-CoV S. D. Further structural comparison of SARS-CoV-2 RBD and SARS-CoV RBD revealed a more obvious structural difference in segment 2 than in segment 1. E. Sequence alignment of SARS-CoV-2 RBD and MERS-CoV RBD revealed low sequence similarity throughout the RBD region, including segment 2. F. 3D-structural superimposition revealed low structural homology between SARS-CoV-2 S and MERS-CoV S, particularly in the RBD region.

Gene sequence alignment revealed a higher sequence identity (76-78%) of SARS-CoV S with SARS-CoV-2 S than with MERS-CoV S (29-30%). Similar to SARS-CoV S and MERS-CoV S, SARS-CoV-2 S is composed of two subunits, S1 and S2, (**Figure 2A**), which may be responsible for host-receptor binding and membrane fusion, respectively. The β-strand-rich S1 subunit consists of an N-terminal domain (NTD), linker region (L), receptor-binding domain (RBD), and subdomain (SD). The α-helix-rich S2 subunit consists of an upstream helix (UH), fusion peptide (FP), connecting region (CR), heptad repeat (HR), central helix (CH), β-hairpin (BH), transmembrane domain (TM), and a cytoplasmic domain (CP) (**Figure 2A**). The S1 RBDs of SARS-CoV and SARS-CoV-2 mediate viral binding to the host receptor ACE2.

To identify unique sequences in the SARS-CoV-2 S RBD distinct from the SARS-CoV and MERS-CoV S RBDs, sequence alignment, and 3D-structural superimposition analysis were performed. Despite the high similarity of the SARS-CoV-2 and SARS-CoV S RBDs, we found many sequence variations and structural differences in their interfaces with ACE2.

First, based on amino acid sequence alignment with the SARS-CoV S RBD, we identified two unique segments in the SARS-CoV-2 S RBD. Segment 1 (residues 447-469, mRNA: AGAUUGUUAGAACUAAGAUUCCAACCACCAUUAAUAUUAAUGGACAUAUCUAACAAAUCCUUCAGAUUA) and segment 2 (residues 479-503, mRNA: UGACUUUAGAUAGUCCGGCCAUCGUGUGGAACAUUACCACAACUUCCAAAAUUAACAAUGAAAGGAAAUGUUAGU) of the SARS-CoV-2 RBD greatly differed in sequence from the corresponding segments in the SARS-CoV RBD (**Figure 2B**), supporting potential unique functions.

Second, the structural superimposition analysis revealed high structural homology between the S proteins of SARS-CoV-2 and SARS-CoV. However, the structural differences were evident in their RBDs (**Figure 2C**). The RBD region was enlarged to analyze the structures of segments 1 and 2 (**Figure 2D**). Segment 1 located in the telechelic structure of SARS-CoV-2 S was a long loop, whereas in SARS-CoV S, it contained small helices. The flexibility of the loop structure facilitates binding to ACE2 (**Figure 2D**). Segment 2 forming a long flexible loop structure is a key region for recognition and binding of ACE2. The high flexibility of segment 2 allows for great structural variation. Segment 2 in SARS-CoV-2 RBD is a more compact structure than the corresponding segment in SARS-CoV RBD (**Figure 2D**). Given the greater structural variation in segment 2, this segment was selected as the specific target sequence for SARS-CoV-2.

Sequence alignment and structural analysis of SARS-CoV-2 S and MERS-CoV S revealed very low sequence identity and structural homology, especially in the RBD region (**Figure 2E-F**). Segment 2 of the SARS-CoV-2 RBD greatly differs in sequence and structure from the corresponding segment in MERS-CoV RBD (**Figure 2E-F**), further supporting the specificity of this segment.

### The unique segment in the SARS-CoV-2 RBD plays a key role in binding to the ACE2 receptor

Differences in the interfaces of the SARS-CoV-2 and SARS-CoV RBDs interacting with ACE2 were analyzed through molecular docking simulation. The SARS-CoV-2 RBD structure acquired from crystal analysis [SARS-CoV-2 RBD (C)] and the SARS-CoV-2 RBD structure constructed through homology modeling [SARS-CoV-2 RBD (M)] were used in the ACE2 docking simulation. The protein sequences of SARS-CoV-2 RBD (C) and SARS-CoV-2 RBD (M) were identical.

Compared with the SARS-CoV RBD, which is tightly packed against the neighboring protomer's N-terminal domain, the RBD of SARS-CoV-2 is located near the central cavity of the homotrimer, which facilitates conformational transitions and binding to ACE2. Crystal structure analysis showed that human ACE2 possessed a claw-like N-terminal peptidase domain composed of two α-helical lobes, α1 and α2 18. At the interface of SARS-CoV/SARS-CoV-2 with ACE2, the RBD (residues 381-588 in SARS-CoV S and 339-592 in SARS-CoV-2 S) forms a concave surface and anchors the entire receptor-binding loop (residues 424-494 in SARS-CoV RBD and 446-517 in SARS-CoV-2 RBD) to the peptidase domain of ACE2. Within this loop region, 13 residues contribute to the binding of both SARS-CoV and SARS-CoV-2 to ACE2. Among these, 8 residues in the SARS-CoV-2 RBD (Y458, Y462, N496, Y498, G505, T509, G511, and Y514) are identical to the corresponding residues in SARS-CoV RBD (Y436, Y440, N473, Y475, G482, T486, G488, and Y491). The remaining 5 residues differ. The 5 residues Y442, L472, N479, D480, and T487 in SARS-CoV particularly determine its host tropism and the interaction with ACE2 19, 20. The five corresponding residues in SARS-CoV-2 are mutated to L464, F495, Q502, S503, and N510.

**Figure 3:**
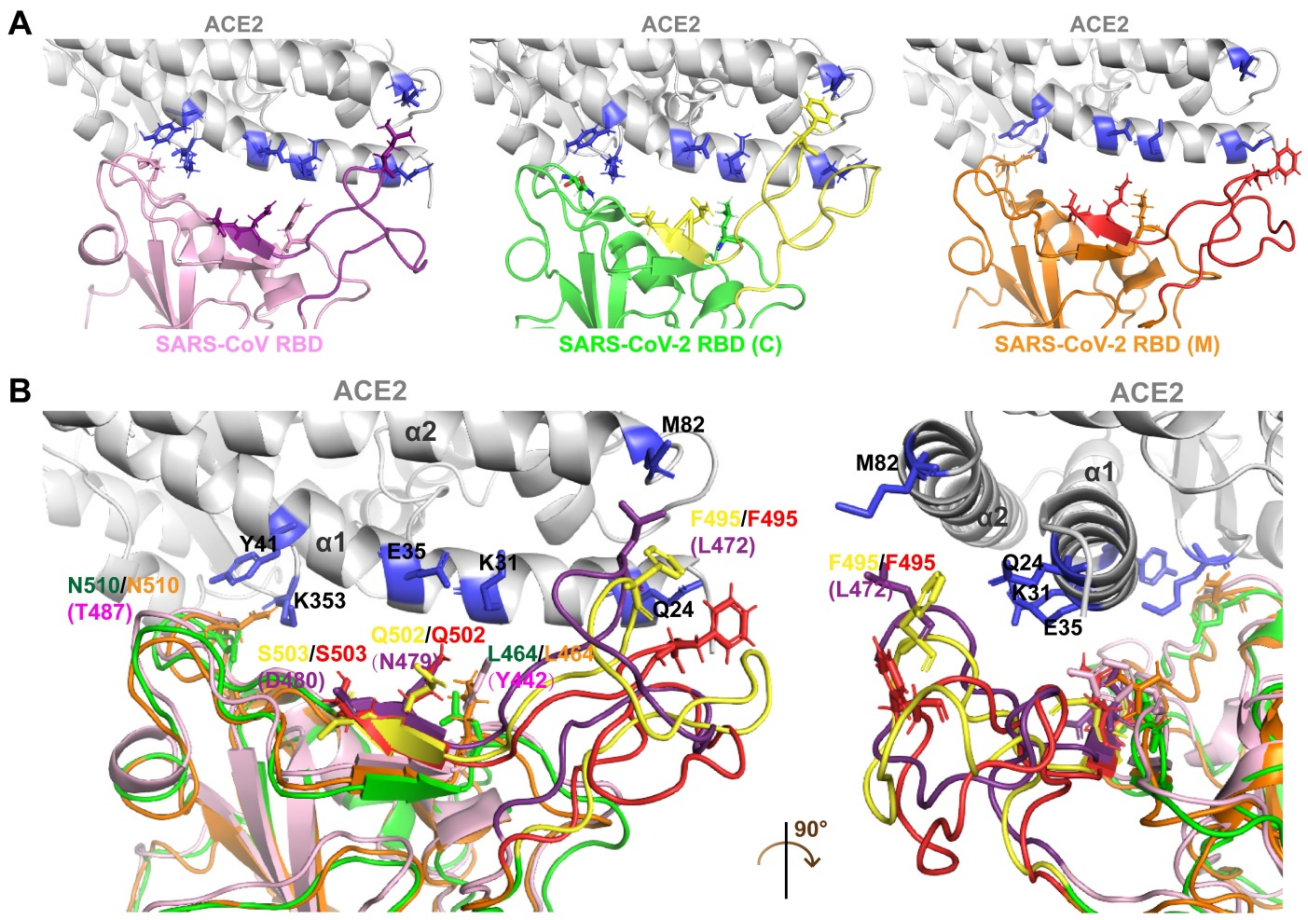
Docking simulation analysis of the S-ACE2 interaction interface. A. Interaction interfaces of ACE2 with the SARS-CoV, SARS-CoV-2 (C) and SARS-CoV-2 (M) RBDs, respectively. The RBDs interact with the α1 and α2 helical lobes of ACE2. Segment 2 in the SARS-CoV, SARS-CoV-2 (C) and SARS-CoV-2 (M) RBDs are indicated in purple, yellow, and red, respectively. B. Structure superimposition revealed that segment 2 contains three key residues interacting with ACE2, including F495 in the SARS-CoV-2 RBD (L472 in the SARS-CoV RBD), Q502 in the SARS-CoV-2 RBD (N479 in the SARS-CoV RBD), and S503 in the SARS-CoV-2 RBD (D480 in the SARS-CoV RBD). There are significant structural differences in segment 2 of the SARS-CoV, SARS-CoV-2 (C), and SARS-CoV-2 (M) RBDs.

Structural comparison of the SARS-CoV and SARS-CoV-2 RBDs revealed an obvious conformational difference in segment 2 (**Figure 3A-B**), which resulted from residue variations. However, the structural differences in segment 2 of the SARS-CoV-2 RBD (C) and the corresponding region in the SARS-CoV-2 RBD (M) resulted from the prediction inaccuracy of homology modeling due to the high flexibility of this segment (**Figure 3A-B**).

Segment 2 in the SARS-CoV-2 RBD includes three critical binding-associated residues, F495, Q502, and S503 (corresponding to residues L472, N479, and D480 in the SARS-CoV RBD) (**Figure 3A-B**). On the one hand, L472 in the SARS-CoV RBD interacts with residue M82 on ACE2 through van der Waals force and supports the viral binding to ACE2. The corresponding residue F495 in the SARS-CoV-2 RBD is even more compatible with the virus-binding hotspot K31 on ACE2 than L472 in SARS-CoV and exhibits stronger van der Waals interaction with M82 on ACE2, providing more support for binding to ACE2. Residue N479 in the SARS-CoV RBD, close to the hotspot K31 in human ACE2, reduces steric and electrostatic interference at the RBD/ACE2 interface and enhances the interaction of SARS-CoV with ACE2. The corresponding residue in SARS-CoV-2, Q502, fits well with K31 in human ACE2 and forms a hydrogen bond with E35 of ACE2. Residue D480 in the SARS-CoV RBD enhances binding to ACE2. The corresponding residue in SARS-CoV-2 RBD, S503, is not as powerful as D480 in SARS-CoV in promoting binding. On the other hand, besides L472, N479, and D480, Y442 and T487 in SARS-CoV also play important roles in ACE2 binding. Residue Y442 in SARS-CoV is not very compatible with K31 in human ACE2, whereas the corresponding residue in SARS-CoV-2, L464, is more compatible with ACE2. Residue T487 in the SARS-CoV RBD, close to the virus-binding hotspot K353, promotes the structural stability of K353 and strengthens viral binding to ACE2. The corresponding residue in SARS-CoV-2, N510, is less compatible with ACE2, although both N510 and T487 are hydrogen-bonded to Y41 on ACE2 (**Figure 3A-B**).

In summary, segment 2 in the SARS-CoV-2 RBD plays a key role in binding to the receptor ACE2. The specificity and critical function of segment 2 suggest that it is suitable for a specific targeting of SARS-CoV-2.

### Design and screening of potentially optimal crRNA candidates that stably bind to Cas13a and viral RNA

**Figure 4:**
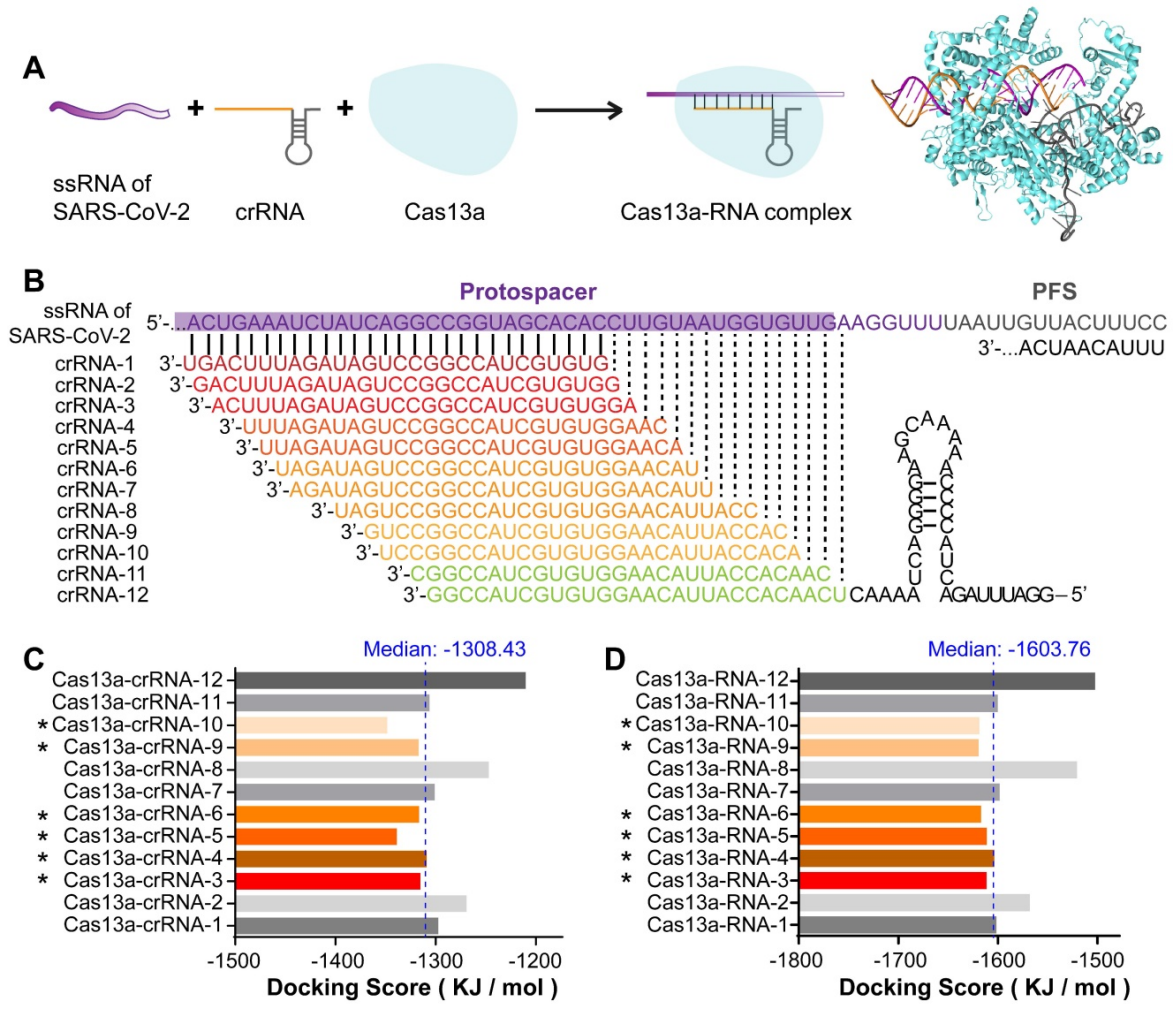
Design and screening of crRNA candidates targeting SARS-CoV-2 RNA. A. Design of crRNAs and Cas13a-RNA docking simulation based on the functional pattern of CRISPR-Cas13a. B. Design of a tiling crRNA library covering segment 2 RNA of SARS-CoV-2. Intervals were set as 1-3 bp. Among the 39 crRNA sequences in the crRNA library, 12 crRNAs with the highest GC content (≥ 0.5) were selected as candidates. C, D. Binding energy analysis of the Cas13a-crRNA complex (C) and Cas13a-RNA (Cas13a-crRNA-target RNA) complex (D). Among the 12 crRNA sequences, six crRNAs with docking scores less than the median, including crRNA-3, 4, 5, 6, 9, and 10, were selected as potentially optimal candidates.

The front-end base sequence fragment of the crRNA is responsible for interacting with Cas13a and ensuring that its cleavage proceeds smoothly. The back-end base sequence of the crRNA is complementary to and binds the target RNA, thereby directing the Cas13a protein (**Figure 4A**).

crRNAs were designed as a tiling library (39 sequences) with 1-3-bp intervals covering the RNA sequence of segment 2 in the SARS-CoV-2 RBD. The sequences and GC contents of the crRNAs are listed in **Table S1** in the Supporting Information. As the GC content is positively correlated with double-stranded DNA or RNA stability, 12 crRNA sequences with the highest GC contents (≥ 0.5) were selected as candidates for subsequent binding simulation (**Figure 4B**). To primarily assess the specificity of these crRNA sequences, they were individually nBLASTed against the NCBI database. The results showed a perfect pairing between the crRNA sequences and SARS-CoV-2 S RNA, indicating the specificity of the crRNA candidates (**Figure S1** in the Supporting Information).

Binding energy analyses were performed to compare the interactions of different crRNA sequences with the target RNA and Cas13a. The docking score was calculated to evaluate the interaction strength. The docking score is negatively correlated with the interaction strength and composite system stability. As shown in **Figure 4C**, the docking scores for the single-stranded crRNAs interacting with Cas13a were low, ranging from -1349.23 to -1211.08 kJ/mol. This may be because the front-end protein-binding base sequences of the crRNAs were similar and could form a hairpin structure embedded in the active cavity of the Cas13a protein, thereby greatly improving the Cas13a-crRNA binding stability. Therefore, differences in the crRNAs binding to Cas13a were mainly caused by different structures of the complementary base sequences of crRNAs. The median docking score of the Cas13a-crRNA complexes was -1308.43 kJ/mol. Among the 12 crRNA candidates, the docking scores for crRNAs 3, 4, 5, 6, 9, and 10 bound to Cas13a were lower than the median, suggesting relatively high Cas13a-guiding potential. The relatively high docking scores of crRNAs 1, 2, 7, 8, 11, and 12 indicated relatively poor binding stability to Cas13a, which may interfere with its activity.

After binding to Cas13a, the crRNA binds to the target RNA through sequence complementarity, thus guiding Cas13a to the target RNA to exert its splicing activity. Therefore, we constructed 3D structures of the double-stranded RNA complexes formed by crRNAs and the target sequence in the S gene. The helical structure of each double-stranded RNA complex was predicted using Make-na server (http://structure.usc.edu/make-na/ server.html). Based on a Cas13a-nucleic acid ligand complex template (PDB ID: 5XWP), a Cas13a-RNA complex was constructed from scratch using a hybrid algorithm of template modeling and free docking. The docking scores for the double-stranded RNAs interacting with Cas13a ranged from -1620.66 to -1503.45 kJ/mol (**Figure 4D**). The median docking score of the Cas13a-double-stranded RNA complexes was -1603.76 kJ/mol. The scores for Cas13a-RNAs 3, 4, 5, 6, 9, and 10 were lower than the median, and those for the others were higher (**Figure 4D**). This suggested that the binding stability of Cas13a-RNAs 3, 4, 5, 6, 9, and 10 was relatively high, likely resulting in a better RNA cleavage effect. Based on the single- and double-stranded RNA docking results, crRNAs 3, 4, and especially 5, 6, 9, and 10 may have the best guiding activity. In summary, the *in-silico* screening indicated that crRNAs 3, 4, 5, 6, 9, and 10 might be potentially optimal crRNA candidates for targeting segment 2 in SARS-CoV-2 S.

### Efficiency of Cas13a-crRNA targeting and cleaving SARS-CoV-2 S

**Figure 5:**
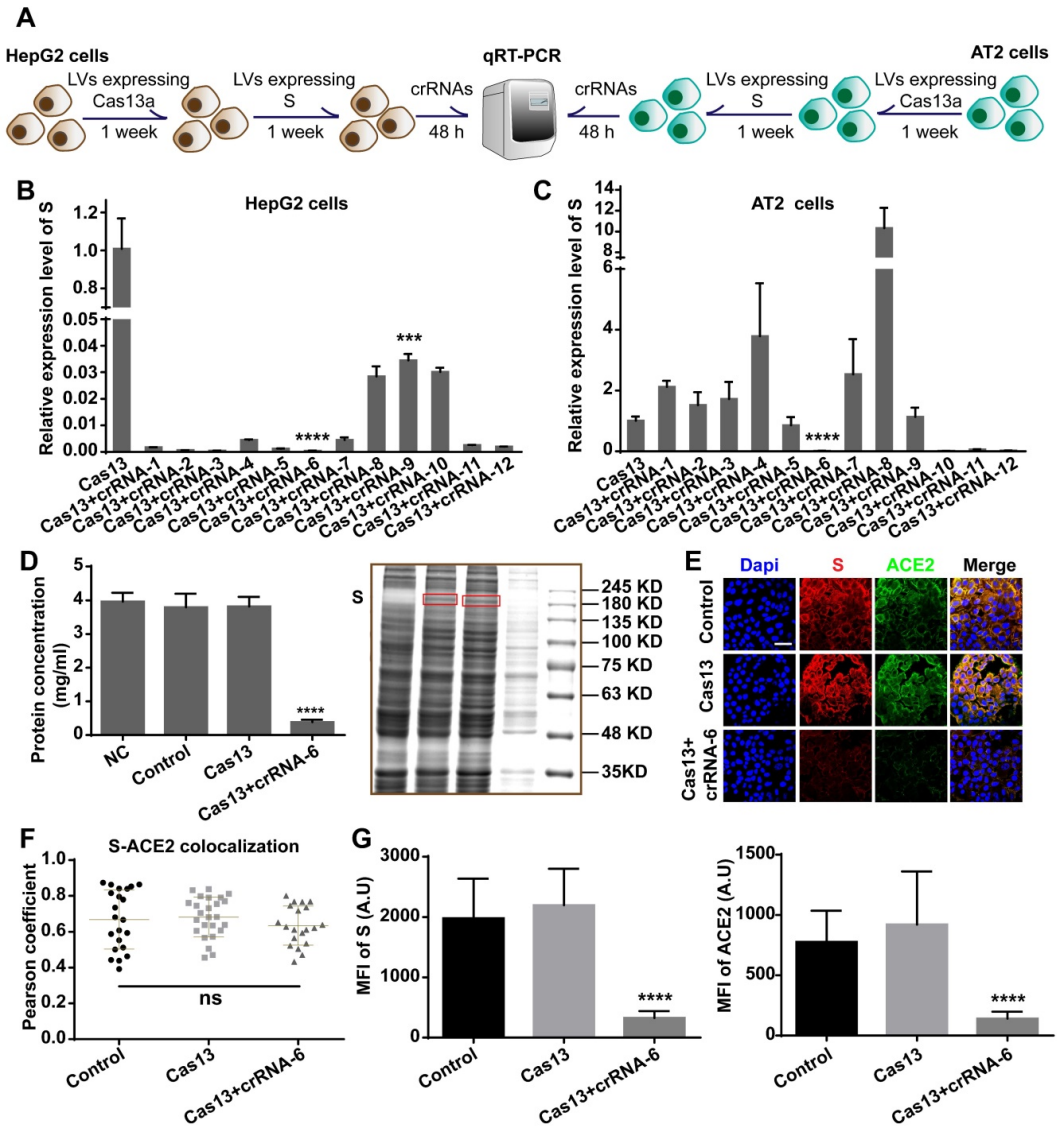
Validation of crRNA efficiency. A. Schematic diagram of the transfection process in HepG2 and AT2 cells for the qRT-PCR assay. B. qRT-PCR analysis showed that in HepG2 cells, crRNA-1-12 induced decreased expression of SARS-CoV-2 S RNA (*P* < 0.001), especially crRNAs 2, 3, 5, and 6. C. qRT-PCR analysis of human AT2 cells showed that crRNAs 6, 10, 11, and 12 induced significantly decreased expression of SARS-CoV-2 S RNA (*P* < 0.0001). D. BCA assay and Coomassie blue staining showed that the total protein concentration in the Cas13+crRNA-6 group was significantly lower than in the NC, Control, and Cas13 groups (*P* < 0.0001). E. Confocal images showing S (red) and ACE2 (green) expression. S and ACE2 colocalized, mainly on the plasma membrane. Scale bar = 50 μm. F. Colocalization of S-ACE2 was quantified and expressed as Pearson coefficient value. There was no significant difference in the colocalization level among the Control, Cas13, and Cas13+crRNA-6 groups (*P* > 0.05). G. Quantification of the mean fluorescence intensity (MFI) in the confocal images showed that S and ACE2 levels were significantly reduced by the Cas13-crRNA-6 system (*P* < 0.0001).

We chose AT2 human alveolar type 2 epithelial cells and HepG2 human hepatoma cells with high ACE2 expression for the S-knockdown assay. The cleavage efficiency was first tested using HepG2 cells and then further evaluated in AT2 cells. HepG2 and AT2 cells were transfected with lentivirus expressing Cas13a, treated with puromycin for 1 week, and then transfected with a plasmid expressing full-length S. After 48 h, the cells were transfected with the crRNAs, followed by total RNA extraction and quantitative reverse-transcription qRT-PCR assay (**Figure 5A**). The qRT-PCR results showed that crRNA-1-12 exhibited different degrees of S RNA-guided cleavage in HepG2 cells. The knockdown efficiencies induced by crRNAs 2, 3, 5, and 6 were the highest (> 99.9%, **Figure 5B**). However, in AT2 cells, crRNAs 6, 10, 11, and 12 exhibited significant cleavage-guiding effects on S RNA, with knockdown efficiencies of >93% (**Figure 5C**). Also, crRNAs 6, 10, 11, and 12 induced collateral cleavage of GFP RNA and Cas13a RNA in AT2 cells (**Figure S2A**-**B** in the Supporting Information).

Together with the binding energy analysis, these results identified crRNA-6 to be a potentially optimal sequence out of the 12 candidates. RNA-denaturing gel electrophoresis showed that ribosomal RNA was cleaved by Cas13a-crRNA-6 in AT2 cells expressing S (**Figure S2C**), providing evidence of the collateral cleavage effect induced by crRNA-6. Moreover, a bicinchoninic acid (BCA) assay, Coomassie blue staining, Western blotting, and immunofluorescence assay were used to quantify the expression of S, ACE2 protein, and total protein in Cas13a-crRNA-6-edited AT2 cells. Equal numbers of AT2 cells (NC group), AT2 cells expressing S (Control group), AT2 cells expressing S and Cas13a (Cas13a group), and AT2 cells expressing S, Cas13a, and crRNA-6 (Cas13a+crRNA-6 group) were lysed with RIPA. The BCA assay showed that the total protein concentration in the Cas13a+crRNA-6 group was reduced by approximately 90% as compared to the Control group (*P* < 0.0001, **Figure 5D**). Equal volumes of lysate samples were separated by SDS-PAGE and stained with Coomassie blue or used for Western blotting. Coomassie blue staining revealed that, compared with the other groups, the Cas13a+crRNA-6 group showed the strongest decrease in total protein (**Figure 5D**), suggesting a robust collateral cleavage effect. Furthermore, the Western blotting analysis indicated that the expression levels of S, ACE2, and GAPDH in the Cas13a+crRNA-6 group were significantly lower than in the Control and Cas13a groups (**Figure S2D**). Confocal imaging revealed strong colocalization of S and ACE2 mainly on the plasma membrane in AT2 cells (**Figure 5E-F**). There was no significant difference in the S-ACE2 colocalization level between the groups (*P* > 0.05, **Figure 5F**). Moreover, S and ACE protein levels were significantly reduced by Cas13a-crRNA-6 (**Figure 5G**). These results suggested that crRNA-6 induced effective and stable cleavage of SARS-CoV-2 S and exhibited a robust collateral cleavage effect.

### Interactions among Cas13a, crRNA-6, and target RNA

**Figure 6:**
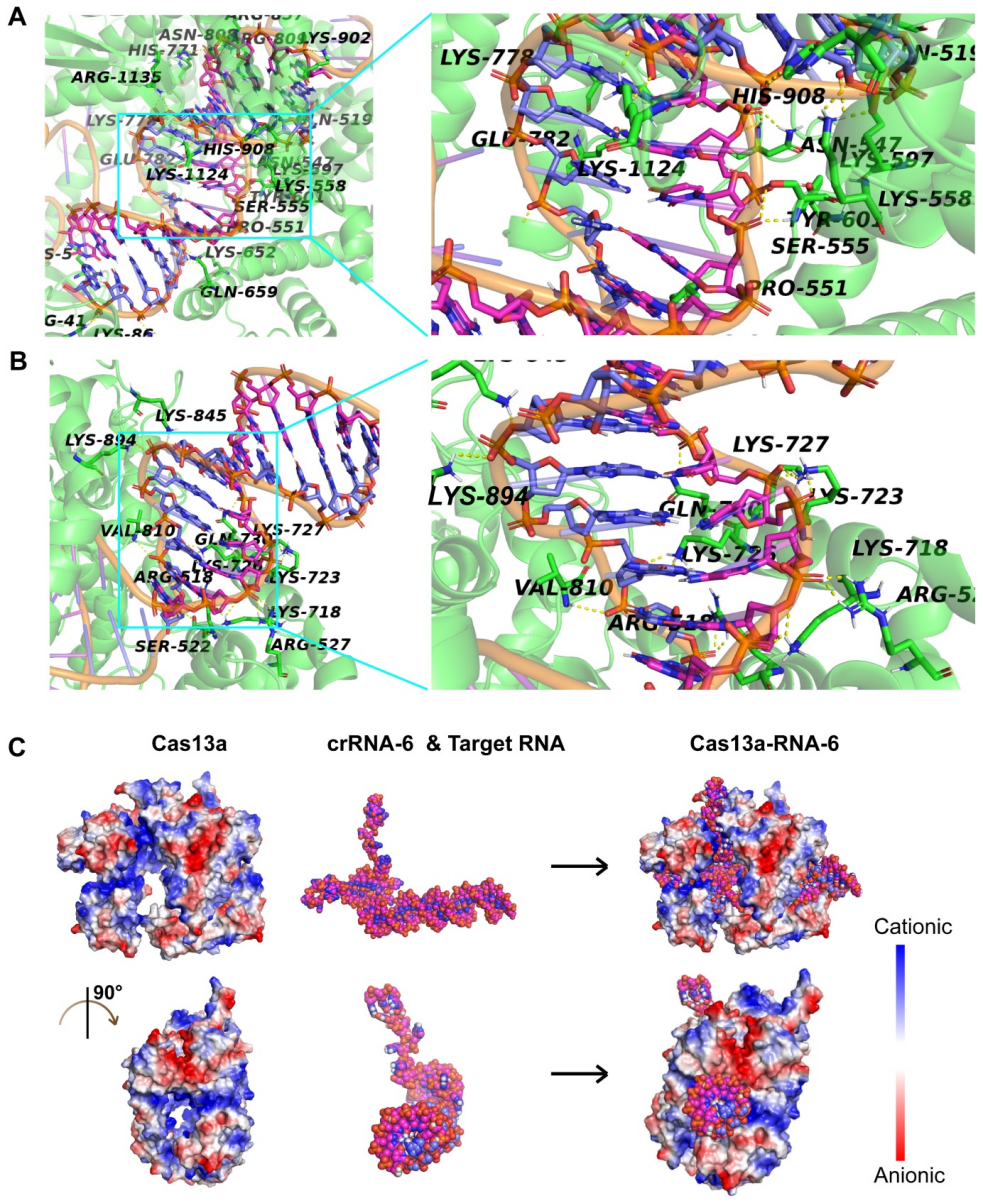
Analysis of the interaction between Cas13a and double-stranded RNA-6 composed of crRNA-6 and target RNA. A. Hydrogen-bond interaction of Cas13a with the bases in the 38-53-bp segment of RNA-6. B. Hydrogen-bond interaction of Cas13a with the bases in the 54-65-bp segment of RNA-6. C. Electrostatic binding surface analysis showed that the positively charged region in the active cavity of Cas13a matches well with the negatively charged surface of RNA-6.

Given that crRNA-6 exhibited the highest guiding activity among all crRNA candidates, a detailed analysis of the interactions among Cas13a, crRNA-6, and target RNA through molecular docking was performed for an in-depth understanding. The double-stranded RNA composed of crRNA-6 and the target RNA was termed RNA-6. The front end of crRNA-6 formed a hairpin-like structure, enabling anchoring of RNA-6 in the active cavity of Cas13a. The key residues in the active site of Cas13a, including S555, T557, H771, R857, K902, and R1135, formed strong hydrogen bonds with the bases in the 38-53-bp segment of RNA-6 (**Figure 6A**). Also, residues Q518, S522, R527, K718, K723, K727, Q730, V810, K845, and K894 of Cas13a, formed strong hydrogen bonds with the bases in the 54-65-bp segment of RNA-6 (**Figure 6B**). Furthermore, K5, R41, N547, S555, K558, K652, N808, R857, K1124, and R1135 interacted with the bases U47 (crRNA-6) and A28 (target RNA) through double hydrogen bonds. All critical hydrogen bonds between Cas13a and RNA-6 are listed in **Table S2** in the Supporting Information. The hydrogen bond interactions were distributed throughout the guide region, enhancing the binding stability of RNA-6 to Cas13a and thus improving the crRNA-guided cleavage activity. Further analysis of the electrostatic binding surface of the Cas13a-RNA-6 complex showed that most regions in the active cavity of Cas13a were positively charged (**Figure 6C**). The negatively charged surface of RNA-6 due to the presence of oxygen atoms resulted in its stable binding to the positively charged region in the active cavity of Cas13a due to good electrostatic matching, further enhancing the crRNA-guided cleavage effect (**Figure 6C**).

### dCas13a-crRNA6 reduces S protein expression without inducing a collateral cleavage effect

Through the guidance of crRNA, dCas13 targets and binds specific RNAs. Due to the loss of endonuclease activity, dCas13 does not cleave target or non-target RNAs. Instead, as a specific RNA-binding protein, dCas13 conjugated with fluorescent proteins, has been applied in RNA imaging and tracking studies 21, 22. In this study, we found that dCas13a-crRNA6 reduced S protein expression without altering its RNA expression level. We performed RNA-denaturing gel electrophoresis to detect whether collateral cleavage could be induced in target cells by dCas13a-crRNA6. As compared with Cas13a-crRNA6 which induced collateral cleavage in AT2-S cells (**Figure S3A**), dCas13a-crRNA6 did not cause the collateral cleavage effect in target cells (**Figure S3B**). qRT-PCR analysis showed that dCas13a-crRNA6 did not alter RNA levels of S and GFP compared with the Control group (*P* > 0.05, *P* > 0.05, **Figure S3C**). Yet S and GFP RNA levels decreased significantly in Cas13a+crRNA6 group compared to the Control group (*P* < 0.0001, *P* < 0.0001, **Figure S3C**). However, immunofluorescence results showed a reduced S protein expression level by dCas13a-crRNA6 (*P* < 0.001, **Figure S3D-E**) whereas the ACE2 (the receptor for S) protein level was not significantly altered (*P* > 0.05, **Figure S3D-E**). These results suggested that dCas13a-crRNA6 resulted in decreased S protein expression without altering the S RNA level in target cells. Moreover, dCas13a-crRNA6 did not induce collateral cleavage effect in target cells. The effect of dCas13a-crRNA6 on S protein expression may be attributed to interference with the target protein translation.

We performed the CCK-8 assay to examine the influence of Cas13a/dCas13a-crRNA6 on cell viability. The 0 h represented the time point for crRNA6 transfection and the first time point for CCK-8 detection. Compared with AT2-S and AT2-S+Cas13, cell growth of AT2-S+Cas13+crRNA6 was significantly lower after 48 h (48 h, *P* < 0.05; 72 h, *P* < 0.001; 96 h, *P* < 0.0001, **Figure S3F**). Cas13a alone did not influence the proliferation of target cells (*P* > 0.05, **Figure S3F**), suggesting that the toxicity of Cas13a-crRNA6 to infected target cells probably resulted from collateral cleavage effect, and may be utilized to kill the infected cells at the early stage of infection. However, dCas13a-crRNA6 did not affect growth of the target cells (*P* > 0.05, **Figure S3G**). It is possible that dCas13a may function as a translation inhibitory protein to reduce expression level of key viral proteins.

### Lack of significant toxicity of Cas13a-crRNA6 in non-target cells

The toxicity of Cas13a-crRNA6 to target cells provided a potential stategy for killing infected cells, however, it raised concerns about whether the off-target effect could occur in non-target cells. To study the effect of Cas13a-crRNA6 on non-target cells, cell growth and collateral cleavage was analyzed using CCK-8 assay and RNA-denaturing gel electrophoresis. There was no significant difference in the proliferation between wild-type AT2 and AT2+Cas13a+crRNA6 cells (*P* > 0.05, **Figure S4A**). Similarly, no significant growth difference was detected between wild-type HepG2 cells and HepG2+Cas13a+crRNA6 cells (*P* >0.05, **Figure S4B**). In contrast with AT2/HepG2+Cas13a+crRNA6 cells, the proliferation of AT2/HepG2-S+Cas13a+crRNA6 cells was inhibited significantly after 48 h (**Figure S4B**), indicating that the Cas13a-crRNA6-induced cytotoxicity may occur only in target cells, which enhanced the confidence about its safety. Furthermore, RNA-denaturing gel electrophoresis showed that in AT2 or HepG2 cells, Cas13a-crRNA6 did not induce collateral cleavage (**Figure S4C**), whereas Cas13a-crRNA6-induced collateral cleavage was evident in AT2/HepG2-S cells (**Figure S4C**). Thus, there was no significant effect of Cas13a-crRNA6 on non-target cells.

### Cas13a-crRNA6 did not target and cleave SARS-CoV S

To further confirm the specificity of crRNA6 targeting SARS-CoV-2 S, we detected the efficiency of Cas13a-crRNA targeting and cleaving SARS-CoV S. qRT-PCR analysis and RNA-denaturing gel electrophoresis were performed to evaluate the RNA expression level of SARS-CoV S and detect whether the collateral cleavage effct was induced in AT2 cells expressing SARS-CoV S by Cas13a-crRNA6. The SARS-CoV S was represented as S' to distinguish from SARS-CoV-2 S. As shown in **Figure S5A**, qRT-PCR analysis showed that the Cas13a-crRNA6 reduced RNA expression level of S (SARS-CoV-2 S) in AT2 cells expressing S (*P* < 0.0001). However, Cas13a-crRNA6 did not alter expression level of S' (SARS-CoV S) in AT2 cells expressing S' (*P* > 0.05). It suggested the Cas13a-crRNA6 did not cleave RNA of S'. Moreover, the RNA-denaturing gel electrophoresis showed that Cas13a-crRNA6 cannot induce collateral cleavage effect in AT2 cells expressing S' (**Figure S5B**). These results indicated that crRNA6 cannot target SARS-CoV S. Instead, it targeted SARS-CoV-2 S and led to the cleavage of SARS-CoV-2 S by Cas13a. It reflected the specificity of crRNA6 targeting SARS-CoV-2 S.

## Discussion

**Figure 7:**
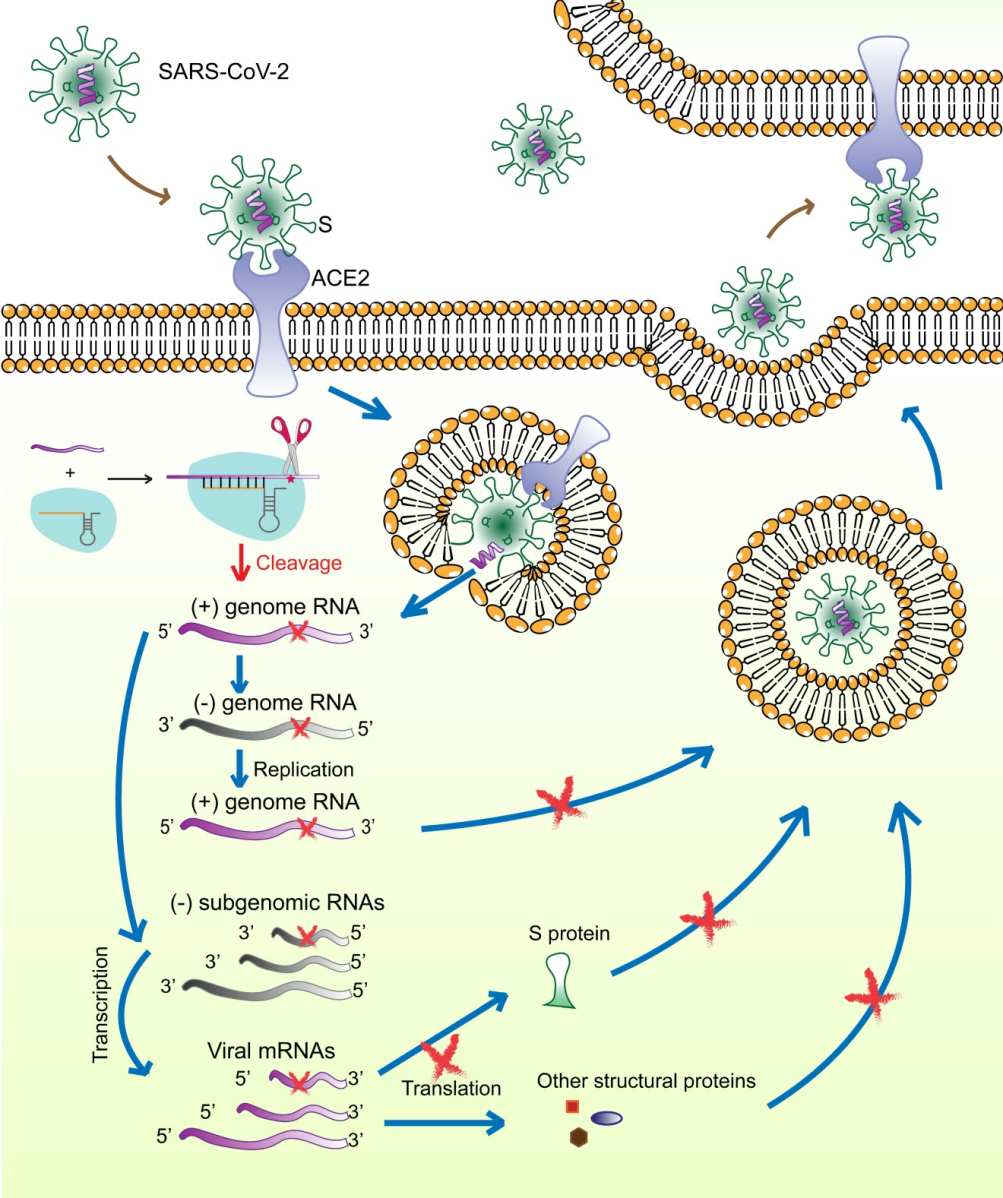
Schematic diagram showing the life-cycle of SARS-CoV-2 and the hypothetical action mechanism of the CRISPR-Cas13a system on SARS-CoV-2. The spike protein (S) binds to the host cell receptor ACE2, followed by membrane fusion and viral genome (positive-sense RNAs) release into the cytoplasm. The subgenomic RNAs are transcribed and serve as templates for mRNA synthesis. These mRNAs are then translated to generate viral structural and accessory proteins. The full‑length positive‑strand genomic RNAs are transcribed to produce full-length negative-sense genomic RNAs as templates for synthesizing new viral genome copies. The virions are assembled from the structural proteins and positive-sense genomic RNAs and are finally released outside the cell. The CRISPR-Cas13a system is activated upon binding to the target viral RNA and extensively degrades viral genomic RNAs and mRNAs, which could be used to effectively reduce viral load.

The surface S protein of SARS-CoV-2 binds to the host cell receptor ACE2, followed by membrane fusion and viral genome (positive-sense RNA) release into the cytoplasm. The subgenomic RNAs are transcribed and serve as templates for mRNA synthesis that are translated to generate viral structural and accessory proteins, including S, M, E, N, and RNA‑dependent RNA polymerase. The full‑length positive‑strand genomic RNAs are transcribed to produce full-length negative-sense genomic RNAs as templates for the synthesis of new viral genome copies. The virions are assembled from the structural proteins and positive-sense genomic RNAs and are finally released outside the cell (**Figure 7**).

Several therapeutic strategies against SARS-CoV-2 are being studied, including preventing viral entry into human cells, interfering with viral protein and RNA synthesis, and preventing virion assembly and release. The spike glycoprotein (S) is of particular interest as a therapeutic target because of its critical role in the virus-host cell interaction. More importantly, according to SARS-CoV-2 mutation data acquired from the National Genomics Science Data Center (NGDC, https://bigd.big.ac.cn/ncov/ variation/annotation), S is one of the regions exhibiting a relatively low mutation frequency. The RBD in S is a popular target for the design of neutralizing antibodies, inhibitors, and vaccines 23, 24. However, drugs against single target of ssRNA-viruses have limited effect. Thus, tools that can precisely identify virus-specific RNA and destroy the entire virus genome are needed. Our previous work had demonstrated, for the first time, CRISPR-Cas13a-induced collateral cleavage in human cells 10. Given the minimal off-target effects and the collateral cleavage effect, the CRISPR-Cas13a system can be applied as a highly efficient and specific RNA knockdown tool to eliminate viruses in mammalian cells. Specifically, once SARS-CoV-2 enters the host cells, viral RNAs and structural proteins are synthesized for assembly and release of viral particles. The Cas13a-crRNA6 complex inside the cell is activated upon binding to target viral RNA and extensively degrades viral genomic RNAs and mRNAs, resulting in failure of viral RNA replication, viral protein synthesis, and virion assembly (**Figure 7**). We hypothesize that the CRISPR-Cas13a system may effectively reduce the viral load after infection. However, due to the limited length of the crRNA, the specific targeting site of the crRNA is critical for accurate guiding. Therefore, identifying the specific segment in the viral genome and designing and screening for crRNAs specifically targeting the viral RNA would contribute to the targeting efficiency of CRISPR-Cas13a.

Here, we highlight an antiviral design pipeline that allows the rapid identification of a virus-specific target site and screening of an optimal crRNA sequence. Unlike the pan-coronavirus inhibition strategy used by Abbott et al. 25, we focused on developing a customized antiviral approach specifically targeting SARS-CoV-2. Although SARS-CoV-2 has a large genome, the S RBD region was targeted because of its critical role in viral invasion into human cells. To identify a highly specific segment within the S RBD of SARS-CoV-2, sequence alignment and structural comparison of the RBDs of SARS-CoV-2 and its close relatives, SARS-CoV and MERS-CoV, were performed. Following the identification of such a specific segment, we designed a crRNA library specifically targeting this segment. To expedite the process and save time, without testing a large number of potential crRNA sequences, we adopted a series of bioinformatics methods to narrow down the candidates. Finally, the efficiencies of crRNA candidates were tested in human HepG2 and AT2 cells, and crRNA-6 was identified as the optimal crRNA sequence that indeed exerted a robust cleavage effect on S and a potent collateral cleavage effect. In addition, the specificity of crRNA6 was confirmed because Cas13a-crRNA6 can not target and cleave S of SARS-CoV.

One of the main advantages of this study lies in the sequence- and structural specificity-based rapid identification of a unique segment in the SARS-CoV-2 structural protein. It is noteworthy that this strategy can be employed without using crystal structure and, instead, using homology modeling from scratch, implying that this approach can be used even in the absence of the precise structures of viral proteins.

Cas13 is potentially useful in both the diagnosis and treatment of viral infection. However, most studies focused on its diagnostic function. CRISPR-Cas13a has been developed as a diagnostic tool, SHERLOCK, for detecting RNA viruses, including the Zika and dengue viruses 9. Similarly, the Cas13-based detection tool, Combinatorial Arrayed Reactions for Multiplexed Evaluation of Nucleic acids (CARMEN), enables robust testing of diverse viruses, including SARS-CoV-2 and influenza A 26. Although this is not the first study to use the CRISPR-Cas13 platform to target SARS-CoV-2, our research is innovative because we provide a rapid design and screening roadmap for Cas13a-based antiviral tools.

Although our research mainly focused on a rapid design and screening pipeline for crRNA targeting the spike protein of SARS-CoV-2, it provides two potential therapeutic strategies using CRISPR-Cas13. One strategy, which aims to eliminate the viruses but simultaneously causes some injury and even death to infected cells, is more suitable for early infection. During the life-cycle of SARS-CoV-2 in human cells, a large number of viral RNAs and proteins exist within the infected cells, which are potential viral targets to be eliminated. The therapeutic value of CRISPR-Cas13a against SARS-CoV-2 may not be limited to a simple knockdown of a specific segment of viral RNA, for example, S RNA. Instead, upon recognizing the target viral RNA by crRNA, Cas13a is activated, cleaving not only target RNA but also non-target RNAs. However, due to the specificity of the Cas13a activation, the effect of CRISPR-Cas13a is limited to target cells, that is, the infected cells. This approach eliminates viruses in infected cells and extensively degrades RNAs. Certainly, RNAs from the infected host cells may also be degraded, resulting in injury or death of these infected cells. In the early stages of infection, complete elimination of the viral genome and eradicating infected cells is an acceptable therapeutic strategy.

The other strategy aims to specifically reduce the production of key viral proteins to interfere with certain key links of virus invasion, viral genome replication or virion assembly. This approach is suitable for late infection. Generally, dCas13 is utilized in RNA imaging and tracking. In addition, dCas13 fused with the catalytic domain of human adenosine deaminase acting on RNA 2 (hADAR2d) has been applied in programmable single-base RNA editing 11. Herein, we found that dCas13-crRNA6 could reduce S protein expression without affecting its RNA level, probably by inhibiting its translation. Also, dCas13-crRNA6 did not induce cytotoxicity caused by the collateral cleavage effect. It also reflected the high specificity of crRNA-6 targeting S. However, this approach against a single target cannot completely eliminate the intracellular viruses. Since we cannot acquire live virus or pseudovirus of SARS-CoV-2, the anti-whole-viral efficacy still needs to be tested in the future.

The biggest barrier to the* in-vivo* application of the CRISPR-Cas platform is its safe and effective delivery. There are several optional delivery carriers, such as viral vectors, lipid nanoparticles, engineered peptides, and polymers. Viral vectors including adeno-associated virus, adenovirus, and lentivirus show high delivery efficiency and stable expression 27, 28; however, their limited loading capacity and potential carcinogenic risk restrict their clinical application. Nanoparticle-based vectors show good penetration ability due to their small sizes, but their packaging and localization efficiencies need to be improved 29. We have previously reported engineered multistage delivery nanoparticles achieving tumor-targeted delivery of CRISPR/dCas9 system 30. In the future, we plan to develop safe and effective delivery vectors for the CRISPR-Cas13a system to achieve *in-vivo* RNA editing.

In summary, we reported a rapid design pipeline for a CRISPR-Cas13a-based anti-viral tool against SARS-CoV-2. As a powerful RNA-editing system, CRISPR-Cas13a has application potential in treating diverse RNA virus infections in mammals.

## Methods

### Sequence alignment

The genome and amino acid sequences of SARS-CoV-2 S, SARS-CoV S, and MERS-CoV S were acquired from the GenBank database of the National Center for Biotechnology Information (NCBI, http://www.ncbi.nlm.nih.gov/). The accession numbers of SARS-CoV-2, SARS-CoV, and MERS-CoV are MN908947.3, AY278488.2, and KF600652.1, respectively. Gene structure maps were drawn with Illustrator for Biological Sequences. Gene sequence alignments were generated and visualized using ClustalW v.2.1 and Mega, respectively. Structural domain prediction of SARS-CoV-2 S protein was conducted using pfam (http://pfam.xfam.org/search/sequence). Protein sequence alignments were carried out and visualized using ClustalW v.2.1 and Exprint 2.0, respectively.

### Homology modeling

Homology modeling of SARS-CoV-2 S was performed using the I-TASSER Server for Protein 3D Structure Prediction (http://zhanglab.ccmb.med.umich.edu/I-TASSER/). The protein structure of S was optimized using Gromacs 4.6.2 software.

### Molecular docking simulation

Besides the structure constructed using homology modeling, S structures obtained from crystal structure analyses were also used in the ACE2 docking simulation. The crystal structures of SARS-CoV-2 S, SARS-CoV S, and MERS-CoV S were acquired from the PDB database (https://www.rcsb.org/). The PDB IDs of SARS-CoV-2 S are 6VXX (2.80 Å), 6LZG (2.5 Å), and 6M0J (2.45 Å). The PDB ID of SARS-CoV S is 6NB6 (4.20 Å). The PDB ID of MERS-CoV S is 5X59 (3.7 Å). The crystal structure of Cas13a-crRNA-target RNA ternary complex was also acquired from PDB (ID: 5XWP). The docking simulation was carried out using ZDock. Among all possible spatial conformations and interaction patterns, the conformation with the lowest energy was selected for visual analysis using PyMol v1.60.

### Cell culture

HepG2 human hepatoma cells were obtained from the American Type Culture Collection (Manassas, Virginia, USA) and were cultured in Dulbecco's modified Eagle's medium (DMEM) supplemented with 10% fetal bovine serum (FBS), 2 mM L-glutamine, and 1% penicillin-streptomycin at 37 °C under 5% CO_2_. AT2 human alveolar epithelial type II cells were purchased from Biobaiye (Shanghai, China) and were cultured in DMEM/F-12 containing 10% FBS, 10 ng mL^-1^ keratinocyte growth factor and 1% penicillin-streptomycin at 37 °C under 5% CO_2_. The medium was changed every other day.

Lentiviruses containing Cas13a were synthesized by GENECHEM (Shanghai, China). Lentiviruses containing S and GFP were acquired from IBSBIO (Shanghai, China). Lentivirus vector maps are provided in **Figure S6**. AT2 cells were transfected with lentiviruses expressing Cas13a at a multiplicity of infection of 10, then with lentiviruses expressing S and GFP. Subsequently, cells were treated with puromycin for 7 days.

### Design and transfection of crRNAs targeting a specific segment in RBD

crRNA sequences were designed using the CRISPR RNA-Targeting Prediction and Visualization Tool (http://bioinfolab.miamioh.edu/CRISPR-RT). The docking scores of Cas13a-crRNA-RNA complexes were calculated to screen potential optimal crRNA candidates. The crRNAs were synthesized by Integrated Biotech Solutions (Shanghai, China). DNA templates for crRNA were produced by PCR using the T7-flanked primers. The reaction mixture was denatured by heating at 95 °C for 5 min followed by snap-chilling on ice for 10 min, and primer extension was carried out by incubation at 72° C for 30 min. The T7 PCR product was then transcribed *in vitro* using the T7 sgRNA MICscript^TM^ KIT (Biomics Biotech, Jiangsu, China). Transcribed crRNA was purified using the EzOmicsTM RNA Quick Clear Kit (Biomics Biotech, Jiangsu, China) according to the manufacturer's instruction. Restriction digestion maps of the crRNAs are listed in **Figure S7**. The crRNA was transfected into cells using Lipofectamine R3000 (Invitrogen, CA, USA).

### qRT-PCR

For each sample, cells were lysed using TRIzol reagent (Invitrogen). Total RNA was isolated as described 10 and was transcribed into cDNA using a reverse transcription kit (RR047A; TaKaRa, Japan). qPCRs were run using SYBR Green Master Mix (Life Technologies) in a DNA Engine Opticon 2 Two-Color qRT-PCR detection system (Bio-Rad Laboratories, Hercules, CA). Target gene expression was normalized to the level of *GAPDH* mRNA. The following primers were used: S-F, GGCTGCGTTATAGCTTGGAATT; S-R, GTGGGTTGGAAACCATATGATTG; GFP-F, CCGCATCGAGAAGTACGAGG; GFP-R, GCGGATGATCTTGTCGGTGA; GAPDH-F, TGCACCACCAACTGCTTAGC; GAPDH-R, GGCATGGACTGTGGTCATGAG; Cas13a-F, TGGAAAAGTACCAGTCCGCC; Cas13a-R, TCGAAGTCCT CGGTCACTCT; S'-F, TGGTATGTTTGGCTCGGCTT; S'-R, GCAGCAAGAAC CACAAGAGC.

### RNA-denaturing gel electrophoresis

For each sample, 5 µg of total RNA was separated on a denaturing 1% agarose gel at 80 V. Gels were imaged using the G:BOX F3 system.

### Cell viability assay

Cell proliferation was measured using the CCK-8 assay. Cells were seeded into 96-well plates at a concentration of 2,000 cells/well. At 0, 24, 48, 72 and 96 h, cells were incubated in a medium containing 10% CCK-8 solution at 37 °C for 2 h. Subsequently, the optical density (OD) value for each well was measured at 450 nm using a microplate reader (BioTek Synergy^tm^ 2; Vermont, USA).

### Confocal microscopy

At 48 h after treatment with crRNA-6, AT2 cells expressing S and Cas13a were harvested for immunofluorescence analysis. Briefly, cells were fixed in 4% paraformaldehyde, permeabilized with 0.5% Triton X-100, and blocked with 5% BSA. The cells were incubated with primary antibodies SARS-CoV-2 spike antibody (GTX632604; GeneTex, USA) and anti-ACE2 antibody (SAB3500346; Sigma-Aldrich) at 4 °C for 16 h. After three washes with PBS, the cells were incubated with Alexa Fluor 594- and Alexa Fluor 633-conjugated secondary antibodies (Life Technologies, USA) at 25 °C for 1 h. The cells were washed thrice with PBS and mounted with ProLong^TM^ Gold Antifade Mountant with DAPI (#P36930, Life Technologies). Images were captured using a fluorescence confocal microscope (Olympus, Japan).

### Coomassie blue staining and Western blotting

Equal numbers of AT2 cells, AT2 cells expressing S, AT2 cells expressing S and Cas13a, and AT2 cells expressing S, Cas13a, and crRNA-6 were lysed with RIPA buffer (R0010; Solarbio, Beijing). Total protein concentrations of the cell lysates were determined using a BCA assay. Twenty microliters of each sample were separated by 10% SDS-PAGE and the gels were stained with Coomassie brilliant blue R-250 Dye (20278; Thermo Fisher Scientific, USA) according to the manufacturer's instructions. The gels were imaged using the FluorChem M imaging system (ProteinSimple, USA). For Western blotting, the proteins were transferred onto polyvinylidene fluoride membranes, followed by blocking in 5% BSA at 25 °C for 2 h. The membranes were incubated with primary antibodies against SARS-CoV-2 spike (GTX632604, GeneTex, USA), ACE2 (SAB3500346, Sigma-Aldrich, USA) and GAPDH (60004-1-Ig, Proteintech, USA) at 4 °C for 16 h, washed with TBST, and incubated with HRP-conjugated secondary antibodies. After three washes with TBST, the blots were visualized using enhanced chemiluminescence.

### Statistical analysis

Data are presented as means ± standard deviations (SDs) from at least three independent experiments. Means (>2 groups) were compared using one-way ANOVA and Dunnett's post-hoc test. *P* < 0.05 was considered significant.

## Supplementary Material

Supplementary figures and tables.
