# Supplementary material for: Rapid design and development of CRISPR-Cas13a targeting SARS-CoV-2 spike protein
Source: Theranostics. 2021 Jan 1;11(2):649–64. doi: 10.7150/thno.51479 (PMC7738867; doi:10.7150/thno.51479)
Supplement: Supplementary file 1 — Supplementary figures and tables. [file thnov11p0649s1.pdf]

Supplementary materials

Rapid design and development of CRISPR-Cas13a targeting SARS-CoV-2 spike protein

Lin Wang[1,2,a], *Junhu Zhou*[1,2,a], *Qixue Wang*[1,2], *Yunfei Wang*[1,2], *and Chunsheng Kang*[1,2,*]

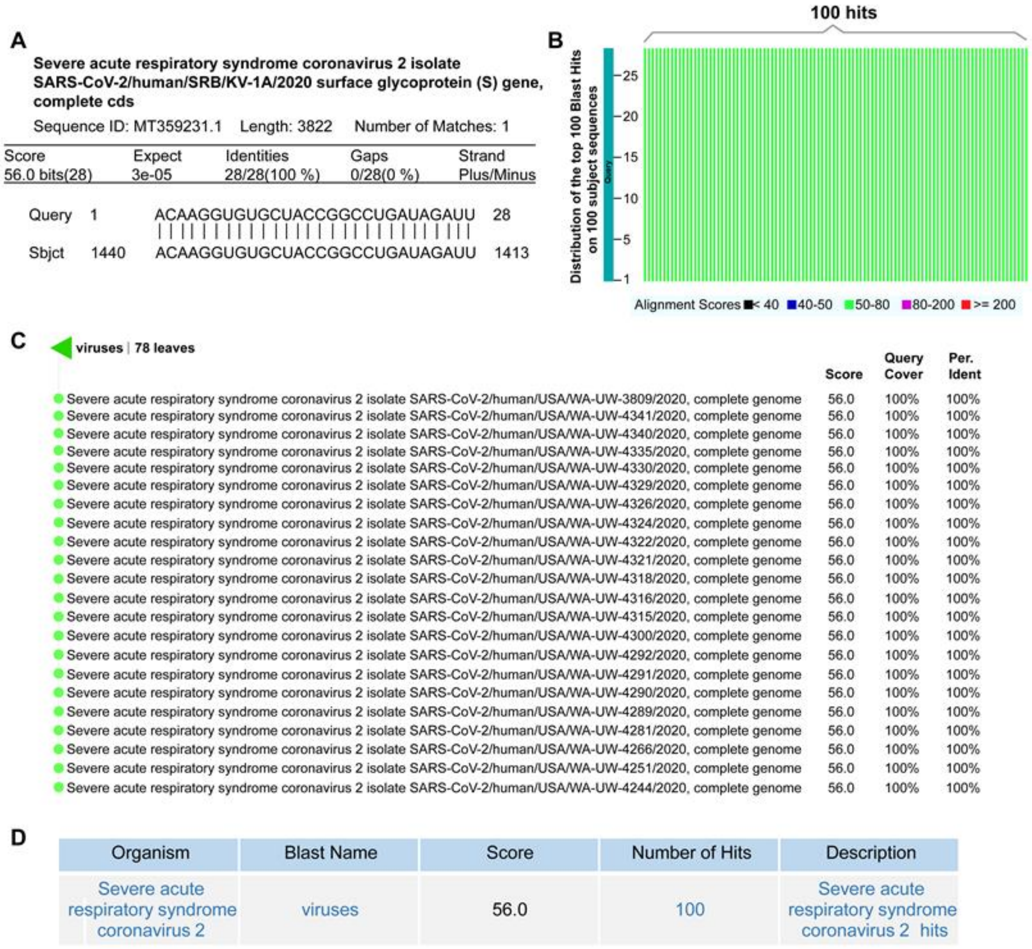

Figure S1. BLAST results of crRNA candidates. A. The crRNA-5 sequence was used as a representative sequence for alignment, revealing perfect pairing between crRNA and SARS-CoV-2 S RNA. B. Distribution of the top 100 BLAST hits on 100 subject sequences. C. BLAST tree view showing the score, query cover, and percent identity of crRNA sequence with hit sequences. D. Taxonomy indicating the specificity of the crRNA candidate targeting SARS-CoV-2.

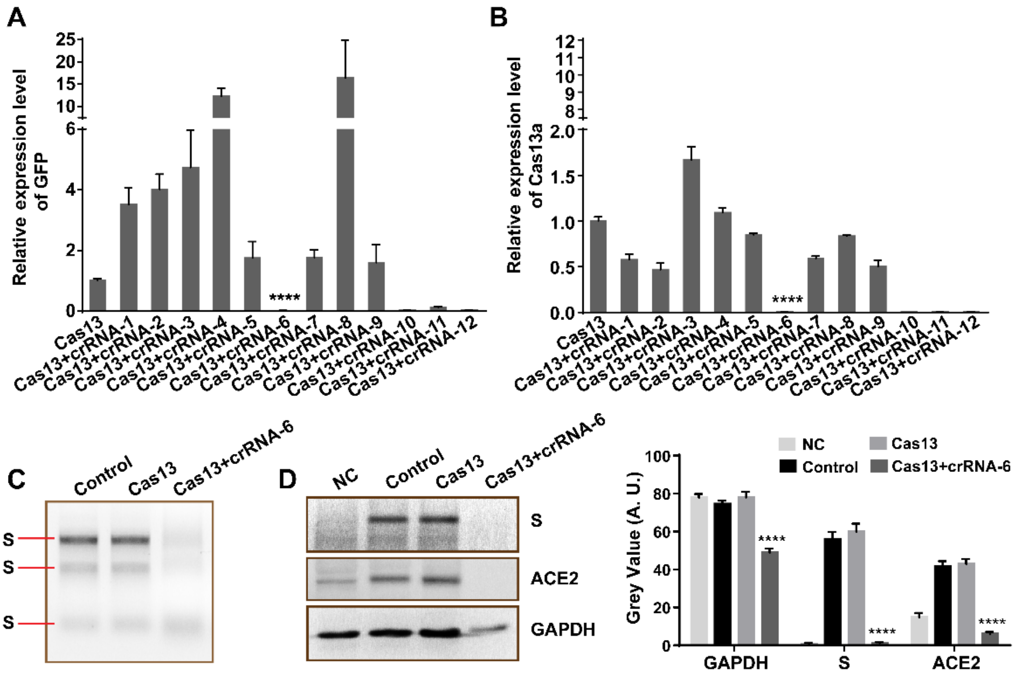

Figure S2. Collateral cleavage induced by crRNA-6. A, B. qRT-PCR analysis showed that crRNAs 6, 10, 11, and 12 reduced GFP (A) and Cas13a (B) RNA expression in AT2 cells (P < 0.0001). C. RNA-denaturing gel electrophoresis showed that ribosomal RNA was cleaved by Cas13–crRNA-6 in AT2 cells expressing S, providing evidence of a collateral cleavage effect induced by crRNA-6. D. Western blots showing decreased levels of S, ACE2, and GAPDH in the Cas13+crRNA-6 group as compared with the Control group (P < 0.0001, P < 0.0001, P < 0.0001).

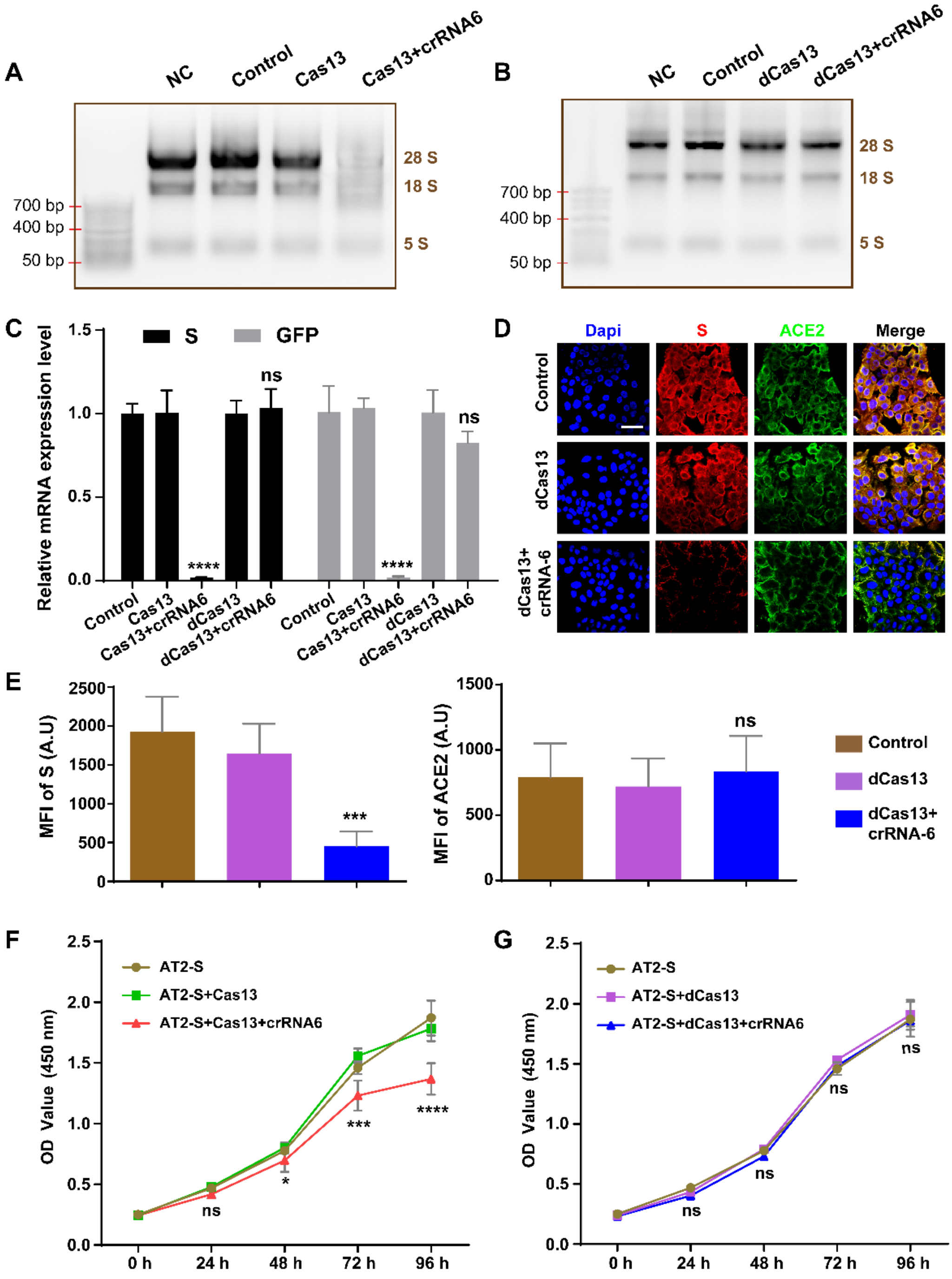

Figure S3. dCas13a-crRNA6 reduced protein expression of S without inducing collateral cleavage effect. A. RNA-denaturing gel electrophoresis showing Cas13a-crRNA6 induced collateral cleavage in AT2-S cells. B. RNA-denaturing gel electrophoresis showing dCas13a-crRNA6 could not induce collateral cleavage effect in AT2-S cells. C. qRT-PCR analysis showing no significant difference in RNA levels of S and GFP between the dCas13a+crRNA6 group and Control group (P > 0.05, P > 0.05). D.

Immunofluorescence imaging showing the expression of S and ACE2 in AT2-S cells (Control group), AT2-dCas13-S cells (dCas13 group), and AT2-dCas13-S-crRNA6 cells (dCas13+crRNA6 group). E. Mean fluorescence intensity (MFI) data showing dCas13a+crRNA6 reduced S protein expression level ($P < 0.001$) without changing ACE2 expression ($P > 0.05$). F. CCK-8 analysis showing Cas13a-crRNA6 inhibited proliferation of AT2-S cells (48 h, $P < 0.05$; 72 h, $P < 0.001$; 96 h, $P < 0.0001$). G. No influence of dCas13a-crRNA6 on of AT2-S cell proliferation ($P > 0.05$).

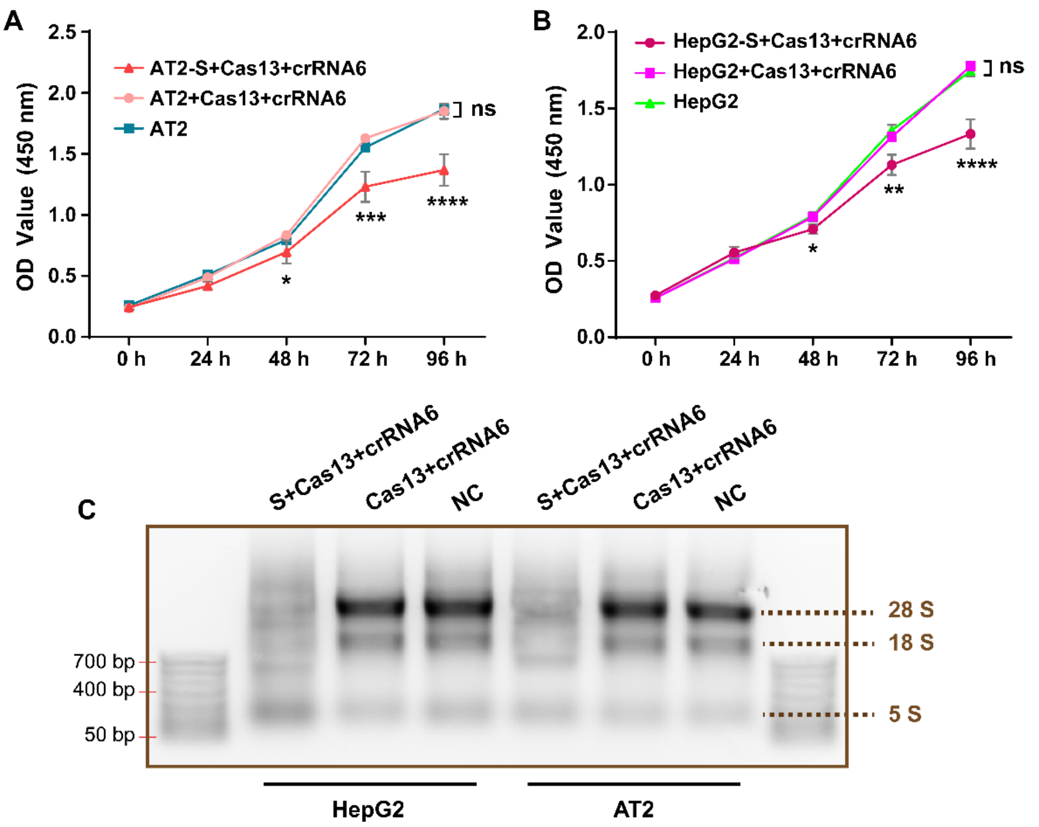

Figure S4. The Cas13-crRNA6 neither influenced the viability of non-target cells nor induced the collateral cleavage effect in non-target cells. A. CCK-8 analysis showing no significant difference in OD values between AT2 and AT2+Cas13+crRNA6 ($P > 0.05$). B. CCK-8 analysis showing no significant difference in OD values between HepG2 and HepG2+Cas13+crRNA6 ($P > 0.05$). C. RNA-denaturing gel electrophoresis showing that Cas13-crRNA6 did not induce collateral cleavage in wild-type HepG2 and AT2 cells.

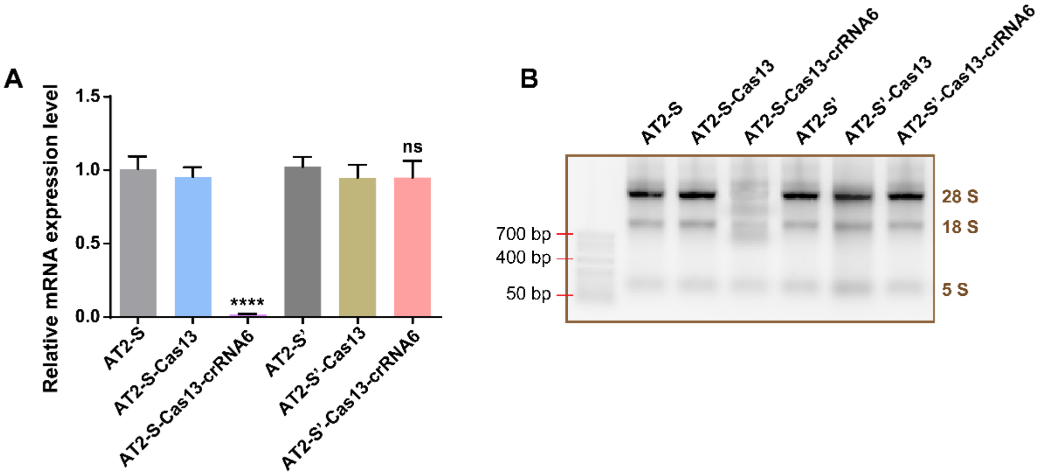

Figure S5. Cas13a-crRNA6 did not cleave RNA of SARS-CoV S. A. qRT-PCR analysis showing Cas13a-crRNA6 did not alter S' (SARS-CoV-2 S) expression level in AT2 cells expressing S' ($P > 0.05$). B. RNA-denaturing gel electrophoresis showing Cas13a-crRNA6 did not induce collateral cleavage effect in AT2 cells expressing S'.

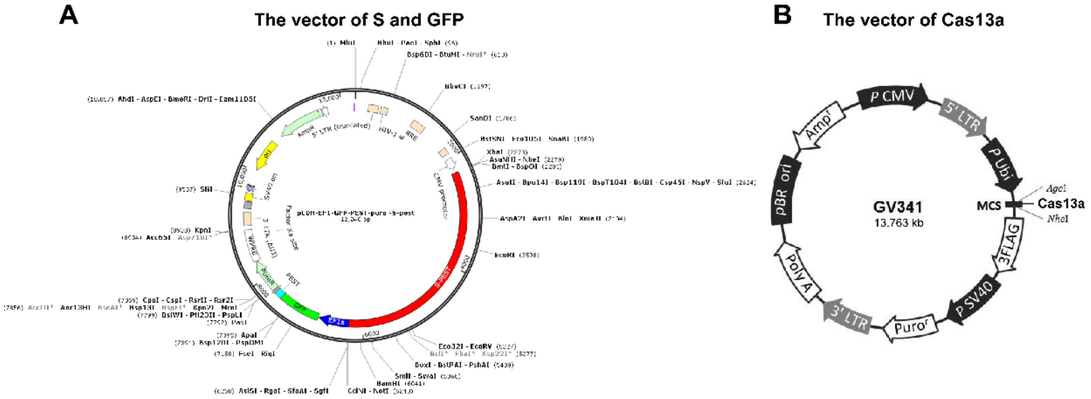

Figure S6. Lentivirus vector maps. A. Vector used for S and GFP. B. Vector used for Cas13a.

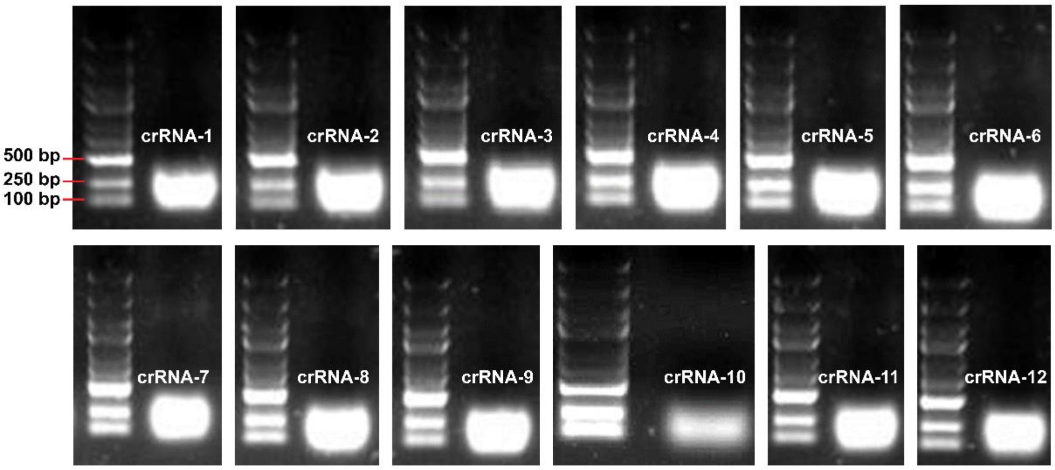

Figure S7. Restriction digestion maps of 12 crRNAs.

Table S1. The crRNA library

No.	Protospacer+PFS	crRNA complementary sequence	crRNA _start	crRNA _end	GC content
1	ACUGAAAUCUAUCAGGCCGGUAGCACACC	UGACUUUAGAUAGUCCGGCCAUCGUGUG	1	29	0.5
2	CUGAAAUCUAUCAGGCCGGUAGCACACCU	GACUUUAGAUAGUCCGGCCAUCGUGUGG	2	30	0.54
3	UGAAAUCUAUCAGGCCGGUAGCACACCUU	ACUUUAGAUAGUCCGGCCAUCGUGUGGA	3	31	0.5
4	AAAUCUAUCAGGCCGGUAGCACACCUUGU	UUUAGAUAGUCCGGCCAUCGUGUGGAAC	5	33	0.5
5	AAUCUAUCAGGCCGGUAGCACACCUUGUA	UUAGAUAGUCCGGCCAUCGUGUGGAACA	6	34	0.5
6	AUCUAUCAGGCCGGUAGCACACCUUGUAA	UAGAUAGUCCGGCCAUCGUGUGGAACAU	7	35	0.5
7	UCUAUCAGGCCGGUAGCACACCUUGUAAU	AGAUAGUCCGGCCAUCGUGUGGAACAUU	8	36	0.5
8	AUCAGGCCGGUAGCACACCUUGUAAUGGU	UAGUCCGGCCAUCGUGUGGAACAUUACC	11	39	0.54
9	CAGGCCGGUAGCACACCUUGUAAUGGUGU	GUCCGGCCAUCGUGUGGAACAUUACCAC	13	41	0.57
10	AGGCCGGUAGCACACCUUGUAAUGGUGUU	UCCGGCCAUCGUGUGGAACAUUACCACA	14	42	0.54
11	GCCGGUAGCACACCUUGUAAUGGUGUUGA	CGGCCAUCGUGUGGAACAUUACCACAAC	16	44	0.54
12	CCGGUAGCACACCUUGUAAUGGUGUUGAA	GGCCAUCGUGUGGAACAUUACCACAACU	17	45	0.5
13	GUAGCACACCUUGUAAUGGUGUUGAAGGU	CAUCGUGUGGAACAUUACCACAACUUCC	20	48	0.46
14	UAGCACACCUUGUAAUGGUGUUGAAGGUU	AUCGUGUGGAACAUUACCACAACUUCCA	21	49	0.43
15	AGCACACCUUGUAAUGGUGUUGAAGGUUU	UCGUGUGGAACAUUACCACAACUUCCAA	22	50	0.43
16	GCACACCUUGUAAUGGUGUUGAAGGUUUU	CGUGUGGAACAUUACCACAACUUCCAAA	23	51	0.43
17	CACACCUUGUAAUGGUGUUGAAGGUUUUA	GUGUGGAACAUUACCACAACUUCCAAAA	24	52	0.39
18	ACACCUUGUAAUGGUGUUGAAGGUUUUAA	UGUGGAACAUUACCACAACUUCCAAAAU	25	53	0.36
19	CACCUUGUAAUGGUGUUGAAGGUUUUAAU	GUGGAACAUUACCACAACUUCCAAAAUU	26	54	0.36
20	ACCUUGUAAUGGUGUUG	UGGAACAUUACCACAAC	27	55	0.32

	AAGGUUUUAAUU	UUCCAAAAUUA			
21	CUUGUAAUGGUGUUGAA GGUUUUAAUUGU	GAACAUUACCACAACUU CCAAAAUUAAC	29	57	0.32
22	UUGUAAUGGUGUUGAAG GUUUUAAUUGUU	AACAUUACCACAACUUC CAAAAUUAACA	30	58	0.29
23	UGUAAUGGUGUUGAAGG UUUUAAUUGUUA	ACAUUACCACAACUUCC AAAAUUAACAA	31	59	0.29
24	GUAAUGGUGUUGAAGGU UUUAAUUGUUAC	CAUUACCACAACUUCCA AAAUUAACAAU	32	60	0.29
25	UAAUGGUGUUGAAGGUU UUAAUUGUUACU	AUUACCACAACUUCCAA AAUUAACAAUG	33	61	0.29
26	AAUGGUGUUGAAGGUUU UAAUUGUUACUU	UUACCACAACUUCCAAA AUUAACAAUGA	34	62	0.29
27	AUGGUGUUGAAGGUUUU AAUUGUUACUUU	UACCACAACUUCCAAAA UUAACAAUGAA	35	63	0.29
28	UGGUGUUGAAGGUUUUA AUUGUUACUUUC	ACCACAACUUCCAAAAU UAACAAUGAAA	36	64	0.29
29	GGUGUUGAAGGUUUUAA UUGUUACUUUCC	CCACAACUUCCAAAAUU AACAAUGAAAG	37	65	0.32
30	GUGUUGAAGGUUUUAAU UGUUACUUUCCU	CACAACUUCCAAAAUUA ACAAUGAAAGG	38	66	0.32
31	UGUUGAAGGUUUUAAUU GUUACUUUCCUU	ACAACUUCCAAAAUUAA CAAUGAAAGGA	39	67	0.29
32	GUUGAAGGUUUUAAUUG UUACUUUCCUUU	CAACUUCCAAAAUUAAC AAUGAAAGGAA	40	68	0.29
33	UUGAAGGUUUUAAUUGU UACUUUCCUUUA	AACUUCCAAAAUUAACA AUGAAAGGAAA	41	69	0.25
34	UGAAGGUUUUAAUUGUU ACUUUCCUUUAC	ACUUCCAAAAUUAACAA UGAAAGGAAAU	42	70	0.25
35	GAAGGUUUUAAUUGUUA CUUUCCUUUACA	CUUCCAAAAUUAACAAU GAAAGGAAAUG	43	71	0.29
36	AAGGUUUUAAUUGUUAC UUUCCUUUACAA	UUCCAAAAUUAACAAUG AAAGGAAAUGU	44	72	0.25
37	AGGUUUUAAUUGUUACU UUCCUUUACAAU	UCCAAAAUUAACAAUGA AAGGAAAUGUU	45	73	0.25
38	GGUUUUAAUUGUUACUU UCCUUUACAAUC	CCAAAAUUAACAAUGAA AGGAAAUGUUA	46	74	0.25
39	GUUUUAAUUGUUACUUU CCUUUACAAUCA	CAAAAUUAACAAUGAAA GGAAAUGUUAG	47	75	0.25

Table S2. Hydrogen-bonds between Cas13a and crRNA-6/viral-RNA

RUN	HBond Residue	BP of RNA	Distance	Angle DHA
1	LYS5	U38:crRNA	2.1	127.3
		U38:crRNA	2.3	124.4
		A28:RNA-SARS-Cov-2	1.9	153.2
2	ARG41	A28:RNA-SARS-Cov-2	2.7	131.4
		A28:RNA-SARS-Cov-2	1.9	158.6
3	LYS86	U27:RNA-SARS-Cov-2	1.8	102.2
4	GLN519	G13:RNA-SARS-Cov-2	1.8	106.2
5	ASN547	U47:crRNA	2.1	124.3
		U47:crRNA	1.6	161.2
		G46:crRNA	2.9	107.1
6	SER555	U45:crRNA	1.7	162.7
		U45:crRNA	2.6	140.3
7	THR557	U47:crRNA	2.8	147.1
8	LYS558	A16:RNA-SARS-Cov-2	1.7	157.8
		U15:RNA-SARS-Cov-2	2.8	99.5
		A16:RNA-SARS-Cov-2	2.3	115.2
9	LYS597	G14:RNA-SARS-Cov-2	1.8	107.2
10	TYR601	G46:crRNA	1.9	145.6
11	LYS652	U24:RNA-SARS-Cov-2	2.3	89
		C23:RNA-SARS-Cov-2	1.9	164.2
12	GLN659	A42:crRNA	2.4	143.8
13	HIS771	U50:crRNA	2.7	112.2
14	LYS778	U47:crRNA	1.6	164
15	GLU782	G46:crRNA	2	146.1
16	ASN808	U50:crRNA	1.9	119.9
		U50:crRNA	2	121.4
17	ARG809	C49:crRNA	2.3	113.1
18	ARG857	A51:crRNA	2.6	121.8
		A51:crRNA	1.5	146.2
		U50:crRNA	2	152.7
		U50:crRNA	2.6	134
		U50:crRNA	1.8	164
19	LYS902	C53:crRNA	2.3	111.9
20	HIS908	G17:RNA-SARS-Cov-2	2.9	158.2
21	LYS1124	C18:RNA-SARS-Cov-2	2	153.1
		C18:RNA-SARS-Cov-2	2.4	114.6
22	ARG1135	A19:RNA-SARS-Cov-2	2	112.6
		C18:RNA-SARS-Cov-2	2.8	109.5
23	GLN518	C12:RNA-SARS-Cov-2	2	139.1
		C12:RNA-SARS-Cov-2	2.1	141.4
24	SER522	C12:RNA-SARS-Cov-2	2.2	121.5

25	ARG527	C56:crRNA	2	129.7
		C56:crRNA	1.5	144
26	LYS718	G55:crRNA	1.8	126.4
		G55:crRNA	2.4	141.8
27	LYS723	C57:crRNA	1.5	146.1
		U58:crRNA	2.4	125.6
28	LYS727	U58:crRNA	1.7	114.8
29	GLN730	G59:crRNA	1.9	132.1
30	VAL810	C11:RNA-SARS-Cov-2	2.8	111
31	LYS845	C7:RNA-SARS-Cov-2	2.5	147.8
32	LYS894	A8:RNA-SARS-Cov-2	2.8	104.3
